# Heat and Pressure Resistance in *Escherichia coli* Relates to Protein Folding and Aggregation

**DOI:** 10.3389/fmicb.2020.00111

**Published:** 2020-02-04

**Authors:** Hui Li, Ryan Mercer, Jürgen Behr, Stephanie Heinzlmeir, Lynn M. McMullen, Rudi F. Vogel, Michael G. Gänzle

**Affiliations:** ^1^Department of Agricultural, Food and Nutritional Science, University of Alberta, Edmonton, AB, Canada; ^2^Institute of Quality Standard and Testing Technology for Agro-Products, Chinese Academy of Agricultural Sciences, Key Laboratory of Agro-food Quality and Safety, Ministry of Agriculture, Beijing, China; ^3^Bavarian Center for Biomolecular Mass Spectrometry, Technical University of Munich, Freising, Germany; ^4^Leibniz-Institute for Food Systems Biology, Technical University of Munich, Freising, Germany; ^5^Technical University of Munich – Lehrstuhl fär Technische Mikrobiologie, Freising, Germany; ^6^College of Bioengineering and Food Science, Hubei University of Technology, Wuhan, China

**Keywords:** locus of heat resistance, heat resistance, protein aggregation, pressure resistance, *Escherichia coli*

## Abstract

The locus of heat resistance (LHR) confers extreme heat resistance in *Escherichia coli*. This study explored the role of the LHR in heat and pressure resistance of *E. coli*, as well as its relationship with protein folding and aggregation *in vivo*. The role of LHR was investigated in *E. coli* MG1655 and the pressure resistant *E. coli* LMM1010 expressing an *ibpA-yfp* fusion protein to visualize inclusion bodies by fluorescence microscopy. The expression of proteins by the LHR was determined by proteomic analysis; inclusion bodies of untreated and treated cells were also analyzed by proteomics, and by fluorescent microscopy. In total, 11 proteins of LHR were expressed: sHSP20, ClpK_GI_, sHSP, YdfX1 and YdfX2, HdeD, KefB, Trx, PsiE, DegP, and a hypothetical protein. The proteomic analysis of inclusion bodies revealed a differential abundance of proteins related to oxidative stress in strains carrying the LHR. The LHR reduced the presence of inclusion bodies after heat or pressure treatment, indicating that proteins expressed by the LHR prevent protein aggregation, or disaggregate proteins. This phenotype of the LHR was also conferred by expression of a fragment containing only sHSP20, ClpK_GI_, and sHSP. The LHR and the fragment encoding only sHSP20, ClpK_GI_, and sHSP also enhanced pressure resistance in *E. coli* MG1655 but had no effect on pressure resistance of *E. coli* LMM1010. In conclusion, the LHR confers pressure resistance to some strains of *E. coli*, and reduces protein aggregation. Pressure and heat resistance are also dependent on additional LHR-encoded functions.

## Introduction

Some strains of *Salmonella* and *Escherichia coli* are extremely resistant to heat and survive thermal treatments that are lethal to a majority of strains in these species (Li and Gänzle, 2016; Mercer R. M. et al., 2017). Heat resistance in *E. coli* and *Salmonella* is conferred by a genomic island termed locus of heat resistance (LHR) (Mercer et al., 2015; Boll et al., 2017). The heat resistance in LHR-positive strains questions the efficacy of thermal pathogen intervention steps that are currently used to ensure food safety (Marti et al., 2016; Ma and Chui, 2017; Mercer R. M. et al., 2017). The LHR occurs in diverse species of beta-Proteobacteria and gamma-Proteobacteria (Gajdosova et al., 2011; Mercer et al., 2015; Lee et al., 2016; Mercer R. M. et al., 2017). It is flanked by mobile genetic elements (Mercer et al., 2015) and is transmitted by lateral gene transfer (Boll et al., 2017). The LHR is present in approximately 2% of strains of *E. coli* but is only rarely present in Shiga-toxin producing strains of *E. coli* (Mercer et al., 2015; Ma and Chui, 2017; Wang et al., 2020). Several anthropogenic environments select for LHR-positive *Enterobacteriaceae*. A high frequency of heat resistant *Enterobacteriaceae* was reported in beef (Dlusskaya et al., 2011; Mercer et al., 2015; Mercer R. M. et al., 2017), in raw milk cheese after thermization at 60°C, and in Daqu, a solid state cereal fermentation that reaches temperatures of up to 60°C (Marti et al., 2016; Boll et al., 2017; Wang et al., 2018). The high frequency of LHR-positive strains of *E. coli* in chlorine-treated waste-water (Zhi et al., 2016) relates to the contribution of the LHR to chlorine resistance (Wang et al., 2020). The presence of the transmissible LHR in pathogenic organisms associated with the food and water supply may present significant challenges to public health.

Putative proteins encoded by the LHR include the heat shock protein sHSP20, the disaggregase ClpK, and a third heat shock protein sHSP_GI_. sHSP20 and ClpK increase heat resistance up to 100 fold in *Pseudomonas* spp. and *Cronobacter* but do not confer heat resistance equivalent to the full length LHR in *E. coli* (Mercer et al., 2015; Mercer R. et al., 2017; Lee et al., 2018). Other genes encoded by the LHR that are required for the full heat resistant phenotypes. These include genes coding for YfdX family proteins with unknown function, and genes which encode for functions predicted to relate to intracellular oxidative stress and accumulation of compatible solutes (Mercer R. et al., 2017). Collectively, LHR-encoded proteins prevent oxidation of cytoplasmic proteins, and oxidation of membrane lipids in *E. coli* (Wang et al., 2020).

Heat shock aggregates cytoplasmic proteins (Strandberg and Enfors, 1991; Hunke and Betton, 2003). Sublethal heat shock at 42°C unfolds or misfolds cellular proteins, which consequently results in protein aggregation if these are not refolded or degraded (Rosen et al., 2002). sHSP20 and ClpK_GI_ bind to misfolded proteins and prevent aggregation, or disaggregate and re-fold the denatured proteins (Lee et al., 2015, 2016, 2018). Functions related to protein homeostasis are also associated with pressure resistance in *E. coli* (Aertsen et al., 2004; Malone et al., 2006; Gänzle and Liu, 2015). Sublethal pressure disassembled inclusion bodies in *E. coli*, and the deletion of genes coding for inclusion body binding proteins IbpA and IbpB reduced the pressure resistance of *E. coli* (Charoenwong et al., 2011). LHR positive and heat resistant *E. coli* are also pressure resistant (Garcia-Hernandez et al., 2015); however, the role of the LHR in pressure resistance of *E. coli* remains unknown. Moreover, the expression and function of genes encoded by the LHR is poorly documented. Therefore, this study aimed to assess the expression of LHR-encoded proteins in *E. coli*, to determine their role in protein aggregation, and to determine whether the LHR mediates cross-resistance to pressure. The expression and cellular localization of proteins was quantified by proteomic analysis in cellular extracts, and in cellular fractions containing inclusion bodies (Jürgen et al., 2001; LeThanh et al., 2005). Protein aggregation was observed with fluorescence microscopy using a strain of *E. coli* that expresses a fusion of *ibpA-yfp* (Govers and Aertsen, 2015; Govers et al., 2016).

## Materials and Methods

### Bacterial Strains and Culture Conditions

Bacterial strains and plasmids that were used in this study are shown in Table 1. *E. coli* MG1655 *ibpA-yfp* and LMM1010 *ibpA-yfp* (Govers and Aertsen, 2015) were grown in Luria Bertani broth (LB, Difco, Sparks, MD, United States) at 37°C. *E. coli* were streaked onto LB agar (Difco) at 37°C, and subcultured at 37°C in LB broth. Plasmids carrying fragments or the full length LHR were extracted from *E. coli* strains DH5α (pRF1), DH5α (pRF2), DH5α (pRF3), DH5α (pRF1-2), and DH5α (pLHR), respectively (Mercer et al., 2015). The plasmids were transformed into *E. coli* MG1655, LMM1010, and MG1655 *ibpA-yfp*. The presence of the plasmids carrying the LHR was confirmed by PCR (Mercer et al., 2015).

**TABLE 1:** Strains and plasmids used in this study.

Strain	Properties	References
*E. coli* MG1655 *ibpA-yfp*	Derivative of *E. coli* MG1655 expressing a IbpA-YFP fusion protein	Govers and Aertsen, 2015
*E. coli* MG1655 *ibpA-yfp* (pRK767), (pRF1), (pRF2), (pRF3), (pRF1-2) or (pLHR)	Derivatives of *E. coli* MG1655 *ibpA-yfp* carrying the plasmid-coded LHR, fragments of the LHR, or the control plasmid pRK767	Govers and Aertsen, 2015; Mercer et al., 2015
*E. coli* LMM1010 *ibpA-yfp*	Derivative of *E. coli* LMM1010 expressing a IbpA-YFP fusion protein	Govers and Aertsen, 2015
*E. coli* LMM1010 *ibpA-yfp* (pRF1); (pRF2), (pRF3), (pRF1-2) or (pLHR)	Derivatives of *E. coli* LMM1010 *ibpA-yfp* carrying the plasmid-coded LHR, fragments of the LHR, or the control plasmid pRK767	Govers and Aertsen, 2015; Mercer et al., 2015
*E. coli* DH5α	Cloning strain for plasmid maintenance	Invitrogen

### Determination of Pressure and Heat Resistance

Pressure resistance of strains of *E. coli* was assessed using stationary-phase cells. Cell suspensions were packed into E3603 tubing (Fisher Scientific, Edmonton, AB, Canada), heat sealed, and placed in a 2.2 mL pressure vessel (Micro-system, Unipress, Warsaw, Poland) filled with bis (2-ethylhexyl) sebacate (Sigma-Aldrich, Germany). The pressure vessel was maintained at 20°C. *E. coli* were treated at 400 MPa; the rate of compression and decompression was 277.8 MPa min^–1^. Temperature changes during compression and decompression did not exceed 4.5°C. Pressure treatments were performed in triplicate independent experiments. Viable cell counts were determined by surface plating on LB agar. Heat resistance was determined by heating strains of *E. coli* at 60 or 70°C for 5 min, followed by immediate cooling on ice and surface plating on LB agar (Dlusskaya et al., 2011). With the initial cell counts about 10^9^ cfu/mL, the cell count of the strain with pRK767 was reduced to below the detection limit, while the cell counts of the strain with pLHR were reduced by less than 3 log (cfu/mL). Cultures were used immediately after heating for microscopic observation of inclusion bodies.

### Isolation of Proteins From Whole Cells and Inclusion Bodies

Inclusion bodies were isolated according to the protocol described by Georgious and Valax (1999). Cells were grown to an OD_600_ 1.0 in 50 mL LB broth, harvested by centrifugation and washed in 5 mL buffer. The cell pellet was resuspended in 10 mM Tris–HC1, pH 7.5, containing 0.75 M sucrose and 0.2 g L^–1^ lysozyme. After 10 min incubation at 20°C, 3 mM EDTA solution was added at a 2:1 (v/v) ratio and cells were kept on ice for approximately 5 min. The cells were lysed by sonication for 3 min, and the lysate was centrifuged at 12,000 × *g* for 30 min at 4°C. The pellet was resuspended in 10 mM Tris–HC1 buffer, pH 8.0, containing 0.25 M sucrose and 1 mM EDTA. The resuspended pellet was layered on the top of a sucrose gradient [40, 53, and 67% (w/w)] in 1 mM Tris–HC1 buffer, pH 8.0, containing 1 mM EDTA. The sucrose gradient was prepared by carefully layering the sucrose solutions. The total volume was 4.7 mL, consisting of 1.3 mL 67% sucrose, 1.2 mL 53% sucrose, and 1.2 mL 40% sucrose solutions and ∼1.0 mL cell lysate. Centrifugation was done at 108,000 × *g* for 90 min at 4°C. Inclusion bodies that were focused in a band at the interface between the 53 and 67% sucrose layers were recovered with a glass pipette. Inclusion bodies were resuspended with native lysis buffer (100 mM Tris pH: 8, 50 mM NaCl, 1 mM EDTA, 1 mM dithiothreitol, 0.1 mM phenylmethylsulfonyl fluoride) and recovered by centrifugation at 10,000 × *g* for 30 min. The pellet was washed again with 1XPBS + 1% TRITON-100, recovered by centrifugation at 30,000 × *g* for 30 min, and dissolved in LDS buffer with 50 mM DTT (Thermo Scientific, Bremen, Germany). Proteins were denatured by heating at 70°C for 10 min and stored at −20°C. Proteome analysis was based on triplicate independent proteins extractions for each condition.

### Protein Digestion and Analysis by LC-MS/MS

Samples representing whole cell extracts or inclusion bodies of pressure treated or untreated cells were alkylated with 55 mM chloroacetamide. Tryptic in-gel digestion was performed from Coomassie-stained protein gels according to standard procedures (Shevchenko et al., 2006). Nanoflow LC-MS/MS was performed with an Eksigent nanoLC-Ultra 1D+ system (Eksigent, Dublin, CA, United States) coupled online to a LTQ-Orbitrap Velos mass spectrometer (Thermo Scientific). Peptides were dissolved in 0.1% formic acid in HPLC grade water, and 1 μg was injected for each measurement. Peptide samples were first loaded on a trap column (75 μm inner diameter × 2 cm, packed in house with 5 μm, Reprosil ODS-3; Dr. Maisch, Ammerbuch, Germany) in 0.1% formic acid. Peptides were transferred to an analytical column (75 μm × 40 cm, C18 column, Reprosil Gold, 3 μm: Dr. Maisch, Ammerbuch, Germany) and eluted at 0.3 μL min^–1^ with a gradient from 96% solvent A (0.1% formic acid and 5% DMSO in HPLC grade water), and 4% solvent B (0.1% formic acid and 5% DMSO in acetonitrile) at 0 min to 68% A and 32% B at 110 min. MS measurement was performed in data-dependent acquisition mode, automatically extracting the ten most prominent precursor ions in the full MS spectra for HCD fragmentation at 30% collision energy. Full MS spectra and MS/MS spectra were acquired at 30,000 and 7,500 resolution, respectively. Dynamic exclusion was set to 60 s.

### Peptide and Protein Identification and Quantification

Label free quantification was performed using MaxQuant (version 1.5.3.30) by searching against an *E. coli* K12 UniProt reference database (version 31.10.2016, 5970 entries) and the 16 LHR protein sequences (Mercer et al., 2015) using the search engine Andromeda with default settings (Cox and Mann, 2008; Cox et al., 2011). Carbamidomethylated cysteine was used as fixed modification; variable modifications included oxidation of methionine and N-terminal protein acetylation. Trypsin/P was specified as the proteolytic enzyme with up to two allowed miscleavage sites. Precursor tolerance was set to 4.5 ppm and fragment ion tolerance was set to 20 ppm. Label-free quantification (Dlusskaya et al., 2011), calculation of intensity based absolute quantification (iBAQ) values and match-between-runs options were enabled and results were filtered for a minimal length of seven amino acids, 1% peptide and protein FDR as well as reverse identifications.

### Visualization of Protein Aggregation

Cell suspensions were transferred to a microscope slide and mounted with a cover slip (Fisher Scientific). Samples were observed with a fluorescence microscope (Carl Zeiss M100, Jena, Germany), and image analysis was performed with AxioVision SE64 (Version 4, Carl Zeiss). The protein aggregation was analyzed by quantifying visible foci in cells, and by enumeration of cells with or without foci. For samples without treatments, the cells were differentiated by the percentages of cells with 0, 1, 2 or >3 protein aggregation foci. For samples treated with pressure or heat, the cells were differentiated by the percentages of cells with or without protein aggregation foci. For each experiment, at least 100 cells were observed and three independent experiments were performed, corresponding to a total of more than 300 cells per sample.

### Statistical Analysis

The number of cells containing none, or one or more foci was determined in triplicate independent experiments and observation of at least 100 cells per experiment. Significant differences between values were determined using one way ANOVA with Holm-Sidak *post hoc* analysis. Significant differences were assessed at an error level of 5% (*P < 0.05).*

The contribution of the LHR or fragments of the LHR on pressure resistance of *E. coli* MG1655 and LMM1010 was determined in triplicate independent experiments. Significant differences among cell counts before and after treatments were determined using one way ANOVA with Holm-Sidak *post hoc* analysis. Significance was assessed at an error probability of 5% (*p* < 0.05).

The experiments of proteomic analysis were performed technical repeats with proteins isolated from quadruplicate independent experiments. MaxQuant results were imported into the MaxQuant associated software suite Perseus (v.1.5.6.0) (Tyanova et al., 2016). The iBAQ intensities were filtered for at least 3 valid values per experimental group. Missing values were imputed from normal distribution (width 0.3, downshift 1.8). Principal component analysis was performed using standard settings in Perseus. A two-sided student’s *t*-test was performed to assess statistical significance. Protein *p*-values were corrected for multiple testing using a permutation based 1% FDR cut-off. Hierarchical clustering was performed on Z-scored median log_2_ transformed iBAQ intensities of significant proteins. Significance was assessed at an error probability of 0.1% (*p* < 0.001).

### Data Deposition

Mass spectrometry data have been deposited to the ProteomeXchange Consortium^1^ via the PRIDE partner repository (Vizcaíno et al., 2013) with the dataset identifier PXD011023.

## Results

### Proteomic Analysis of *E. coli* MG1655 *ibpA-yfp*

**FIGURE 1:**
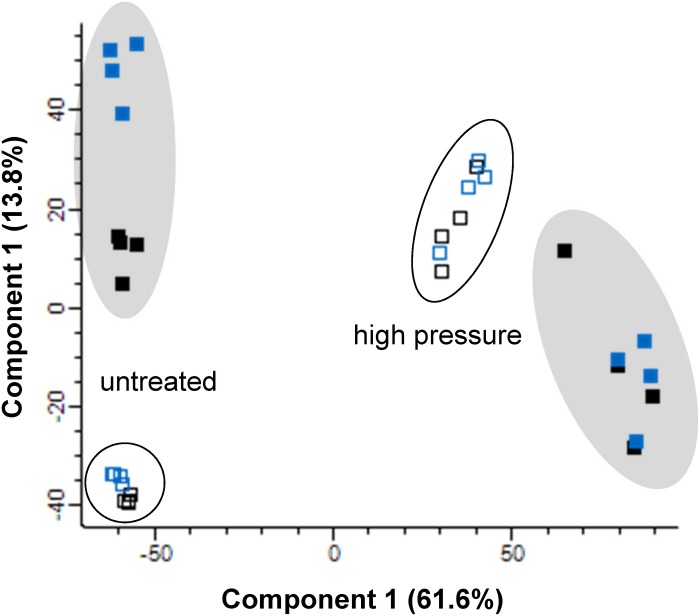
Principle component analysis of proteomic data for inclusion bodies (filled) and whole cell extracts (open) in *E. coli* MG1655 *ibpA-yfp* carrying pLHR (blue) or pRK767 (black). Cells were analyzed without treatment, or after treatment at 400 MPa for 3 min. Proteome analyses were performed in quadruplicate independent experiments with technical repeats for each sample.

Proteomic data was obtained for a strain carrying an *ibpA-yfp* fusion to be able to compare visualization of inclusion bodies with proteome data. Proteome data for inclusion bodies and whole cell extracts in *E. coli* MG1655 *ibpA-yfp* (pRK767) and MG1655 *ibpA-yfp* (pLHR) before and after pressure treatment were initially analyzed by principle component analysis (Figure 1). Samples from untreated and pressure treated cells clustered separately. Samples of inclusion bodies also clustered separately from whole cell extracts obtained from the same samples. Samples prepared from inclusion bodies of untreated *E. coli* MG1655 *ibpA-yfp*(pRK767) clustered separately from *E. coli* MG1655 *ibpA-yfp*(pLHR), indicating that expression of the LHR proteins may influence protein aggregation (Figure 1). Specifically, the small heat shock protein IbpA was overrepresented in inclusion bodies in untreated cells of both *E. coli* MG1655 *ibpA-yfp* (pRK767) (3.4 fold; *p* = 4.0 × 10^–7^) and MG1655 *ibpA-yfp* (pLHR) (6.9 fold; *p* = 9.4 × 10^–7^), validating that isolation of inclusion bodies included the aggregated proteins (Supplementary Table S1). The preparation of inclusion bodies also included membrane proteins (Supplementary Table S1).

### Expression of LHR-Encoded Proteins

**FIGURE 2:**
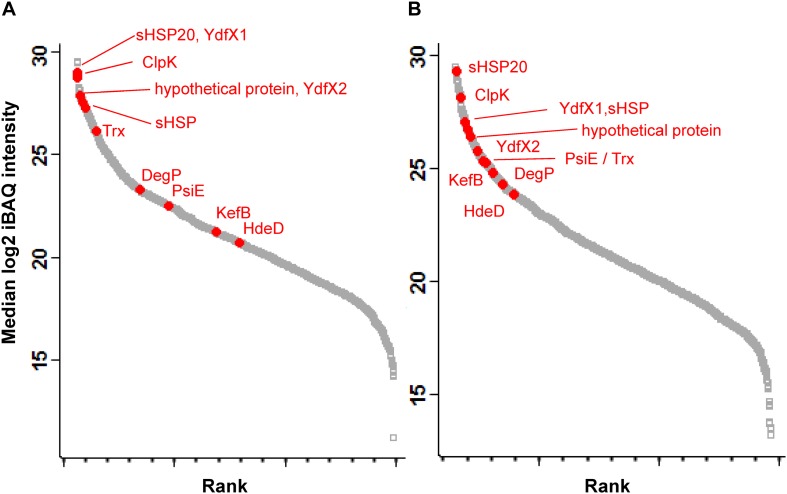
Abundance of proteins expressed in *E. coli* MG1655 *ibpA-yfp* (pLHR). **(A)** Whole cell extracts; **(B)** inclusion bodies. Gray squares: background proteome; red dots: proteins expressed by the LHR. Proteome analyses were performed in quadruplicate independent experiments with technical repeats for each sample.

Eleven proteins encoded by the LHR were detected in *E. coli* MG1655 *ibpA-yfg* (pLHR). All of these 11 proteins were present both in whole cell extracts and inclusion bodies (Figure 2). sHSP20 was among the most highly expressed proteins in both whole cell extracts and inclusion bodies (Figures 2A,B). KefB and the phosphate-starvation-inducible E family protein PsiE had significantly higher abundance in inclusion bodies of *E. coli* MG1655 *ibpA-yfp* (pLHR) compared to the whole cell extract (*p* < 0.001, Figure 2).

### Expression of Proteins in *E. coli* MG1655 *ibpA-yfp* (pLHR) and *E. coli* MG1655 *ibpA-yfp* (pRK767)

**FIGURE 3:**
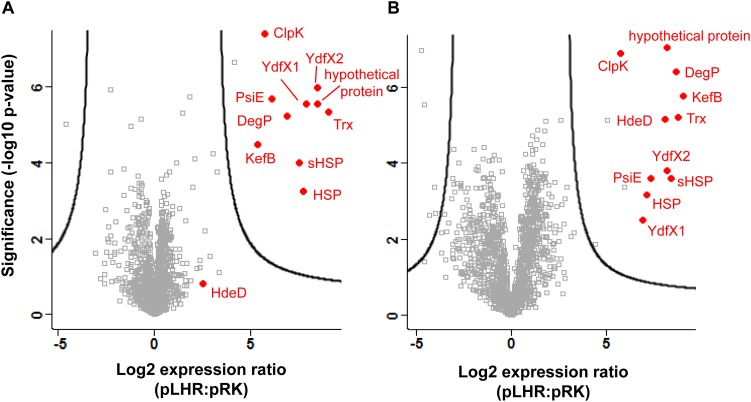
Expression of proteins in *E. coli* MG1655 *ibpA-yfp* carrying pLHR or pRK767. Shown is the log_2_ transformed ratio of the protein abundance in *E. coli* MG1655 *ibpA-yfp* (pLHR) relative to the abundance in MG1655 *ibpA-yfp* (pRK767). **(A)** Expression ratio in whole cell extracts; **(B)** expression ratio in inclusion bodies. The level of significance was evaluated using a two-sided unpaired student’s *t*-test and *p*-values were corrected for multiple testing using a permutation based 1% FDR cutoff and the sample background variability s0. Gray squares: background proteome; red dots: proteins expressed by the LHR. Proteome analyses were performed in quadruplicate independent experiments with technical repeats for each sample.

Protein expression in cell extracts and inclusion bodies was compared between untreated *E. coli* MG1655 *ibpA-yfp* containing pRK767 or pLHR (Figures 3A,B). Three of the 11 LHR-encoded proteins are additionally encoded in the chromosome of *E. coli* MG1655; the disaggregase ClpK, thioredoxin Trx and the potassium/hydrogen exchanger KefB. Expression of these three proteins was at least 30-fold higher in *E. coli* MG1655 *ibpA-yfp*(pLHR) when compared to *E. coli* MG1655 *ibpA-yfp*(pRK767) (Figure 3). Differences in protein expression in whole cell extracts of *E. coli* pRK767 and pLHR related almost exclusively to LHR-encoded proteins (Figure 3A); however, in inclusion bodies of the LHR-negative strain, five additional proteins were enriched (Figure 3B and Table 2). These proteins included an acid phosphatase, a putative deamidase, the glyoxylate/hydroxypyruvate reductase GhrB and peroxiredoxin OsmC. The activities of GhrB and OsmC relate to oxidation-reduction reactions and protect against oxidative stress (Lesniak et al., 2003).

**TABLE 2:** Proteins of inclusion bodies that are overrepresented in *E. coli* MG1655 *ibpA-yfp* (pRK767) compared to *E. coli* MG1655 *ibpA-yfp* (pLHR).

Protein products	Gene	Razor +	Fold change
	names	unique peptides	(pRK767/LHR)
Class B acid phosphatase	*aphA*	4	15.7
Putative reactive intermediate deaminase TdcF	*tdcF*	3	19.3
Glyoxylate/hydroxypyruvate reductase B	*ghrB*	6	26.5
Peroxiredoxin OsmC	*osmC*	6	23.8
Uncharacterized protein YaeQ	*yaeQ*	3	16.3

### Effect of Pressure on the Proteome of *E. coli*

**FIGURE 4:**
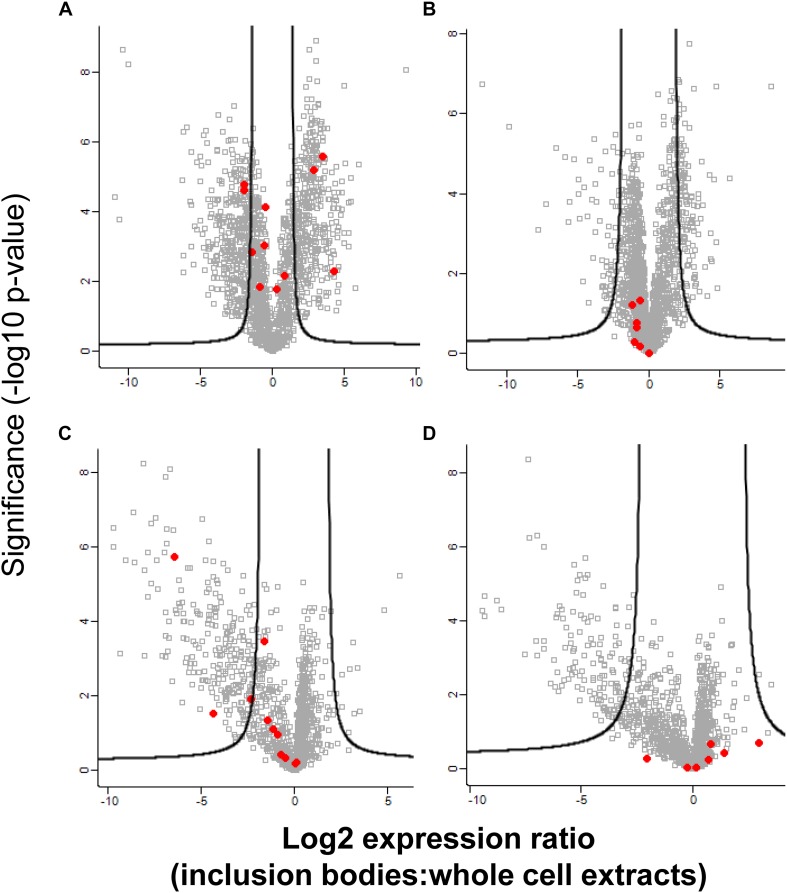
Abundance of proteins in inclusion bodies and whole cell extracts in *E. coli* MG1655 *ibpA-yfp* in inclusion bodies and whole cell extracts, with and without treatment with 400 MPa for 3 min. Shown is the log_2_ transformed ratio of the protein abundance in inclusion bodies relative to the abundance in whole cell extracts obtained from the same sample. **(A)** Expression ratio of untreated *E. coli* MG1655 *ibpA-yfp* (pLHR); **(B)** expression ratio in untreated *E. coli* MG1655 *ibpA-yfp* (pRK767); **(C)** expression ratio in *E. coli* MG1655 *ibpA-yfp* (pLHR) after treatment at 400 MPa; **(D)** expression ratio in *E. coli* MG1655 *ibpA-yfp* (pRK767) after treatment at 400 MPa. The level of significance was evaluated using a two-sided unpaired student’s *t*-test and *p*-values were corrected for multiple testing using a permutation based 1% FDR cutoff and the sample background variability s0. Red dots: proteins expressed by the LHR, other proteins are indicated by gray squares. Note that the *x*-axes for untreated cell **(A,B)** and for pressure treated cells **(C,D)** are scaled differently. Proteome analyses were performed in quadruplicate independent experiments with technical repeats for each sample.

To further explore how inclusion bodies change under stress, the proteomes of *E. coli* MG1655 *ibpA-yfp*(pRK767) and MG1655 *ibpA-yfp*(pLHR) whole cell extracts and inclusion bodies were analyzed after pressure treatment at 400 MPa (Figure 4). Pressure treatment disrupts protein aggregates and bacterial membranes (Gänzle and Liu, 2015; Gayán et al., 2017). In untreated cells, proteome analysis identified proteins that were enriched in the whole cell extract as well as proteins that were enriched in the inclusion bodies (Figures 4A,B). After pressure treatment, very few proteins were enriched in inclusion bodies and most enriched proteins were found in whole cell extracts (Figures 4C,D). In untreated cells, ribosomal proteins were overrepresented in whole cell extracts, while they were overrepresented in inclusion bodies of pressure treated cells (Figure 4 and Supplementary Table S1). Outer membrane proteins were observed in whole cell extracts of untreated cells but these were not present in pressure treated cells (Supplementary Table S1). The small heat shock protein IbpA was overrepresented in inclusion bodies in untreated cells of both *E. coli* MG1655 *ibpA-yfp*(pRK767) and MG1655 *ibpA-yfp*(pLHR), while the level of IbpA was not different in pressure treated cells of the two strains, indicating that pressure treatment dispersed IbpA associated with inclusion bodies.

### Protein Aggregation After Heat and Pressure Treatment

**FIGURE 5:**
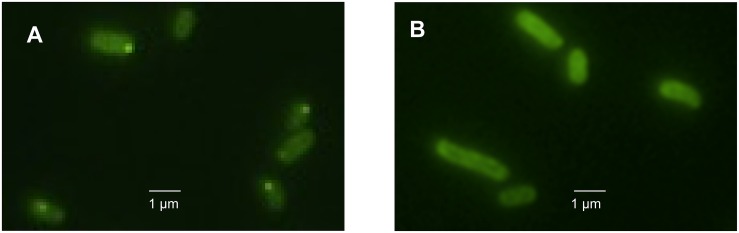
Images of *E. coli* MG1655 *ibpA-yfp* cells harboring pRK767 **(A)** or pLHR **(B)** after treatment of 70°C for 5 min.

To obtain further insight into the influence of the LHR on protein folding and aggregation, inclusion bodies in *E. coli* MG1655 *ibpA-yfp*(pRK767) and MG1655 *ibpA-yfp*(pLHR) were visualized by fluorescence microscopy (Govers and Aertsen, 2015; Govers et al., 2016, 2018). Examples of the images are shown in Figure 5. Experiments also employed *E. coli* LMM1010 *ibpA-yfp*(pRK767) and LMM1010 *ibpA-yfp*(pLHR) (Supplementary Table S3). *E. coli* LMM1010 is a pressure resistant derivative of *E. coli* MG1655 with a higher basal expression from heat shock promotors (Aertsen et al., 2004). Untreated cultures of *E. coli* MG1655 *ibpA-yfp*(pLHR) contained fewer cells with one inclusion body and more cells contained 2 or more foci when compared to *E. coli* MG1655 *ibpA-yfp*(pRK767) (Table 3). After treatment at 60°C or 400 MPa, protein aggregation was reduced in both *E. coli* MG1655 *ibpA-yfp* and LMM1010 *ibpA-yfp* containing pLHR (Table 4 and Supplementary Table S3), demonstrating that the LHR altered stress-induced protein aggregation.

**TABLE 3:** Differential enumeration of cells containing none, one, two, or more than three foci representing protein aggregates in *E. coli* MG1655 *ibpA-yfp* (pRK767) and *E. coli* MG1655 *ibpA-yfp* (pLHR).

Number of protein aggregates (foci) per cell	% of cells of *E. coli* MG1655 *ibpA-yfp* harboring a different number of fluorescent foci	
	
	pRK767	pLHR
0	3.0 ± 1.3	4.8 ± 3.2
1	92.8 ± 2.3	73.5 ± 9.4
2	3.9 ± 2.8	19.6 ± 10.5
>3	0.3 ± 0.6	2.0 ± 0.7

*Data are based on triplicate independent experiments with observation of 100 cells per replicate.*

**TABLE 4:** Differential enumeration of cells containing none, or one or more foci representing protein aggregates in *E. coli* MG1655 *ibpA-yfp* (pRK767) and *E. coli* MG1655 *ibpA-yfp* (pLHR).

	% of cells of *E. coli* MG1655 *ibpA-yfp* with a different number of fluorescent foci	
	
# of foci	pRK767	pLHR
**After 60°C for 5 min**		
1 or more	62.9 ± 5.0	29.8 ± 6.6
None	37.1 ± 5.0	70.2 ± 6.6
**After 400 MPa for 3 min**		
1 or more	77.7 ± 2.9	57.9 ± 4.5
None	22.3 ± 2.9	42.1 ± 4.5

*Shown is the percentage of cells with foci after treatment at 60°C for 5 min or at 400 MP for 3 min. Data are based on triplicate independent experiments with observation of 100 cells per replicate.*

### Contribution of the LHR to Pressure Resistance of *E. coli* MG1655 *ibpA-yfp*

**FIGURE 6:**
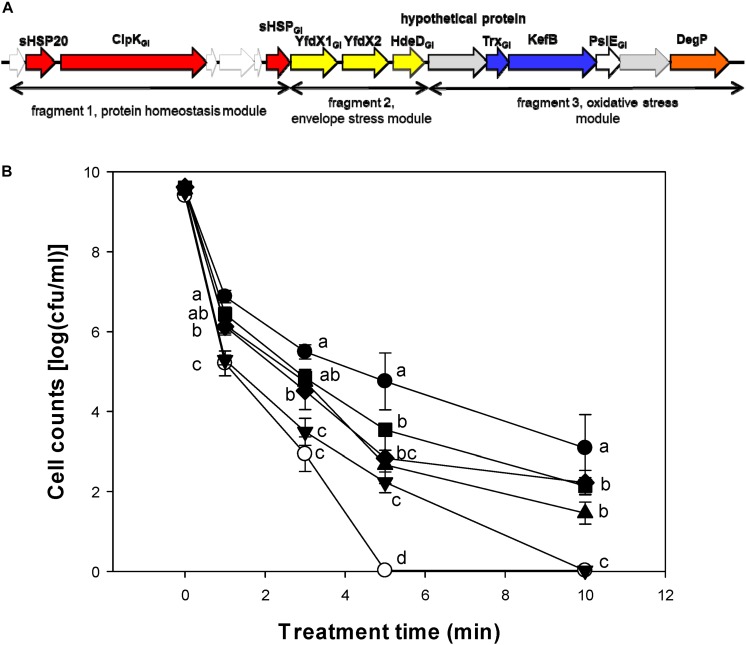
Contribution of the locus of heat resistance (LHR) or parts of the LHR to pressure resistance of *E. coli* 1655 *ibpA-yfp*. **(A)** Schematic representation of the LHR1. Only genes that are expressed in *E. coli* MG1655 are indicated. Proteins are color coded based on their predicted function: red, heat shock proteins; yellow, hypothetical proteins with a possible relationship to envelope stress; blue, genes related to oxidative stress; orange, DegP with possible relationship to signaling in the Cpx, EvgA and σE pathways. Genes carry the footnote “GI” for genomic island if an ortholog of the same gene is present in genomes of *E. coli*. The three fragments of the LHR that were cloned are indicated. **(B)** Pressure resistance of *E. coli* MG1655 *ibpA-yfp* carrying pRK767 (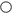) or the derivatives of this vector with the full length LHR (∙), the LHR fragment F1 (▲), F2 (▼), F3 (◆), or F1-2 (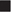). Cells were treated at 400 MPa and 20°C for 1–10 min. Data are shown as means ± standard deviation of triplicate independent experiments. Significant differences between cell counts were determined with one way ANOVA and the Holm-Sidak *post hoc* analysis; values with different superscripts are significantly different (*p* < 0.05).

We also determined whether the LHR or the fragments of the LHR with different contribution to heat resistance (Mercer et al., 2015; Mercer R. M. et al., 2017) contribute to pressure resistance of *E. coli* (Figure 6). After 10 min, viable cell counts of the strain *E. coli* MG1655 *ibpA-yfp*(pRF1) and MG1655 *ibpA-yfp*(pRF3) were higher than that of *E. coli* MG1655 *ibpA-yfp*(pRRK767) (Figure 6B). The highest viable cell counts were observed with *E. coli* MG1655 *ibpA-yfp*(pLHR). Fragment 2 of LHR did not make a major contribution to the pressure resistance of *E. coli* (Figure 6B).

### Effect of Partial Cloning of the LHR on Protein Aggregation *in vivo*

To determine whether those proteins of the LHR that contribute to pressure resistance also mediate the LHR-related protein aggregation, the effect of the LHR fragments on protein aggregation in cells treated at 70°C or 400 MPa was investigated. After heat treatment at 70°C, no protein aggregates were observed in *E. coli* MG1655 *ibpA-yfp*(pLHR) and (pRF1) (Table 5). Pressure treated *E. coli* MG1655 *ibpA-yfp*(pLHR) and (pRF1) had also fewer protein aggregates compared to *E. coli* MG1655 *ibpA-yfp*(pRK767) (Table 5). The protein homeostasis module of the LHR was apparently sufficient to mediate the protein aggregation phenotype that was observed in cells carrying the full length LHR.

**TABLE 5:** Differential enumeration of cells containing none, or one or more foci representing protein aggregates in *E. coli* MG1655 *ibpA-yfp* (pRK767), *E. coli* MG1655 *ibpA-yfp* (pLHR), *E. coli* MG1655 *ibpA-yfp* (pRF1), *E. coli* MG1655 *ibpA-yfp* (pRF2), *E. coli* MG1655 *ibpA-yfp* (pRF1-2), and *E. coli* MG1655 *ibpA-yfp* (pRF3).

	% of cells of *E. coli* MG1655 ibpA-yfp with or without fluorescent foci					
	
Presence of foci	pRK767	pLHR	pRF1	pRF2	pRF1-2	pRF3
**After 70°C for 5 min**						
+	44.7 ± 1.3^a^	0.0^b^	0.0^b^	43.9 ± 3.2^a^	0.0^b^	38.8 ± 11.5^a^
-	55.3 ± 1.3^b^	100.0^a^	100.0^a^	56.1 ± 3.2^b^	100.0^a^	61.2 ± 11.5^b^
**After 400 MPa for 3 min**						
+	77.7 ± 2.9^a^	57.9 ± 4.5^b^	52.0 ± 3.9^bc^	63.5 ± 6.2^ab^	36.7 ± 10.3^c^	66.6 ± 3.3^ab^
-	22.3 ± 2.9^b^	42.1 ± 4.5^ab^	48.0 ± 3.9^ab^	36.5 ± 6.2^b^	63.3 ± 10.3^a^	33.4 ± 3.3^b^

*Shown is the percentage of cells with foci after treatment at 70°C for 5 min or at 400 MP for 3 min. Data are based on triplicate independent experiments with observation of 100 cells per replicate. Values in the same row are significantly different if they do not share a common superscript (*p* < 0.05).*

### Contribution of the LHR to Pressure Resistance of *E. coli* MG1655 and LMM1010

**FIGURE 7:**
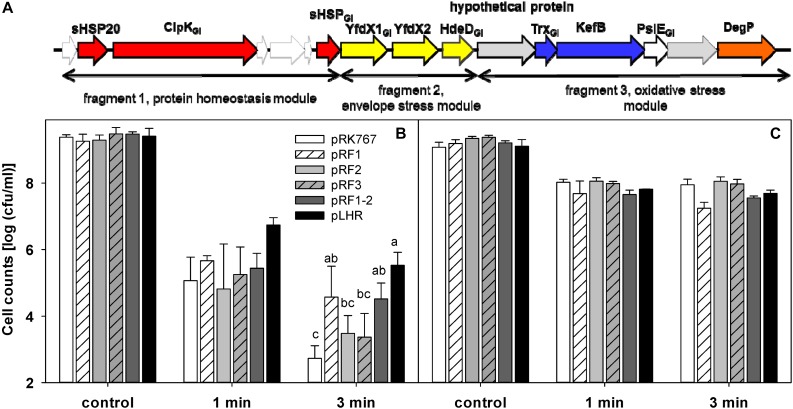
Contribution of partial or full of the locus of heat resistance (LHR) to pressure resistance of the wild type strain *E. coli* MG1655 and its derivative strain *E. coli* LMM1010. **(A)** Schematic representation of the LHR1. For details, see legend to Figure 6. **(B)** Pressure resistance of *E. coli* MG1655 carrying the plasmids indicated. **(C)** Pressure resistance of *E. coli* LMM1010 carrying the plasmids indicated. The strains carrying pRK767 or the derivatives of this vector with the full length LHR or the LHR fragments F1, F2, F3, or F1-2 were treated at 400 MPa at 20°C for 1 or 3 min. Data are shown as means ± standard deviation of triplicate independent experiments. Significant differences between cell counts were determined with one way ANOVA and the Holm-Sidak *post hoc* analysis; values with different superscripts are significantly different (*p* < 0.05).

The effect of the LHR on pressure resistance was also determined with *E. coli* MG1655 and LMM1010 (Figure 7). The effect of pLHR or fragments of the LHR on *E. coli* MG1655 and MG1655 *ibpA-yfp* were comparable (Figure 7B). *E. coli* LMM1010 exhibited much higher pressure resistance than *E. coli* MG1655 and its resistance was independent of the presence of the LHR (Figure 7C). Cell counts of *E. coli* LMM1010 were reduced by only about 1 log (cfu/mL) after treatment at 400 MPa for 3 min; cell counts of *E. coli* LMM1010 *ibpA-yfp* were reduced by about 6 log (cfu/mL) after treatment (Supplementary Figure S1).

The LHR did not increase pressure resistance of *E. coli* LMM1010, a derivative of MG1655, indicating that there are other mechanisms of pressure resistance in *E. coli* LMM1010. Cloning of the LHR into *E. coli* LMM1010 *ibpA-yfp* also had no effect on pressure resistance (Supplementary Figure S1). Although the LHR did not influence pressure resistance of *E. coli* LMM1010 *ibpA-yfp*, the effect of the LHR on protein aggregation in the untreated cells of *E. coli* LMM1010 *ibpA-yfp* (Supplementary Table S2) and in pressure or heat treated cells of *E. coli* LMM1010 *ibpA-yfp* (Supplementary Table S3) was comparable to that observed in *E. coli* MG1655 *ibpA-yfp* (Tables 3, 4).

## Discussion

This study quantified the expression of LHR-encoded proteins, and their differential abundance as soluble proteins and in protein aggregates. Protein aggregation in individual cells was quantified *in vivo* with an *ibpA-yfp* fusion and fluorescence microscopy, and was related to heat and pressure resistance of *E. coli*.

### Abundance of LHR-Encoded Proteins

Mass spectrometry based proteomics (Zhang et al., 2013) was employed to analyze the expression level of LHR-encoded proteins and their differential distribution in inclusion bodies. Wild-type strains of *E. coli* and *Salmonella* carry chromosomal or plasmid-encoded copies on the LHR but both have equivalent effects on heat resistance (Boll et al., 2017; Mercer R. M. et al., 2017), indicating that cloning of the LHR on a low copy plasmid does not alter its physiological function. Of the 16 open reading frames encoded by the LHR in *E. coli* AW1.7 (Mercer et al., 2015), 11 proteins were expressed. These proteins are encoded both in the 15 kbp LHR1 and in the 19 kbp LHR2 (Lee et al., 2015; Mercer et al., 2015; Boll et al., 2017); the proteome analysis thus delineates a core set of proteins that confers heat resistance. LHR-encoded proteins are among the most abundant proteins in *E. coli*; highly expressed proteins include sHSP20, ClpK_GI_, and sHPS, and the proteins with unknown function YdfX1 and YdfX2. Their high expression conforms to the observation that cloning of these proteins increases heat resistance of *Pseudomonas*, *Klebsiella*, and *Cronobacter* (Bojer et al., 2010; Gajdosova et al., 2011; Lee et al., 2018), and that their deletion from the LHR decreases the level of LHR-mediated heat resistance (Mercer R. et al., 2017). The high level of expression of multiple LHR-encoded proteins also supports the conclusion that LHR-mediated heat resistance is a multifactorial phenotype (Mercer R. et al., 2017; Wang et al., 2020). High level protein expression in *E. coli* imposes a substantial metabolic burden on the cell and may lead to metabolic alterations and a reduced growth rate (Pasini et al., 2016). The maintenance of the LHR in a substantial proportion of *E. coli* (Mercer et al., 2015; Lee et al., 2018) demonstrates that some of the environmental niches for these organisms exert a substantial selective pressure for maintenance of the LHR.

### Differential Abundance of Proteins in Inclusion Bodies

Proteome analysis revealed the composition of inclusion bodies in recombinant bacterial cells (Jürgen et al., 2001; LeThanh et al., 2005); we adapted this methodology to explore the connection between expression of the LHR (Mercer et al., 2015) and protein aggregation. The introduction of the *ibpA-yfp* fusion into strains of *E. coli* affected intracellular protein aggregation (Govers and Aertsen, 2015; Govers et al., 2016). Therefore, tool used to assess protein aggregation, the IbpA-YFP fusion protein, influences the measured variable protein aggregation. The use of these strains nevertheless enables comparative analysis of inclusion bodies in *E. coli ibpA-yfp* carrying pLHR or a control plasmid, and additionally enables the study of protein aggregation by microscopic observation. Proteins with differential abundance in *E. coli* MG1655 *ibpA-yfp* (pLHR) and *ibpA-yfp* (pRK767) include the metabolic enzymes AphA and TdcF and the oxidoreductases GhrB and OsmC. The class B acid phosphatase AphA dephosphorylates phosphomonoesters to transfer the phosphate to hydroxyl groups (Thaller et al., 1997); TdcF interacts with toxic 2-ketobutyrate to prevent its accumulation (Burman et al., 2007). The glyoxylate reductase GhrB oxidizes glycolate to glyoxylate as part of the biosynthesis of serine (Nuñez et al., 2001). Glycolate metabolism is also differentially regulated by *E. coli* during hyperosmotic stress (Shabala et al., 2009). Peroxiredoxin OsmC mitigates oxidative stress caused by organic hydroperoxides by detoxification with cysteine thiol groups (Lesniak et al., 2003). The differential composition of inclusion bodies in *E. coli* MG1655 *ibpA-yfp* (pLHR) and (pRK767) suggests that the LHR reduces aggregation of metabolic proteins and stress-related proteins, and conforms to the reduced oxidation of cytoplasmic proteins in strains of *E. coli* carrying the LHR (Wang et al., 2020).

### LHR Effects on Pressure Resistance

Pressure treatment dissociated protein aggregates in *E. coli* MG1655 *ibpA-yfp* (Govers and Aertsen, 2015) and pressure resistance of *E. coli* is enhanced by expression of heat shock proteins including IbpA and DnaK (Aertsen et al., 2004; Malone et al., 2006). We therefore assessed whether LHR-encoded functions increase pressure resistance in *E. coli* MG1655 and LMM1010 (Hauben et al., 1997) and in the *ibpA-yfp* derivatives of both strains. LHR-mediated functions influenced the effects of pressure on protein aggregates but the effect of the LHR on pressure resistance was strain dependent. Resistance of *E. coli* MG1655 was increased by LHR expression but the presence of the LHR did not impact the pressure resistance of *E. coli* LMM1010. The pressure resistant mutant *E. coli* LMM1010 overexpresses heat shock proteins including Lon, ClpX, and DnaK (Aertsen et al., 2004), demonstrating that alternative routes to pressure resistance exist in *E. coli* (Gayán et al., 2019). Pressure resistance of *E. coli* is mediated by multiple proteins including the alternative sigma factors σ^E^ and σ^S^, proteins that protect against oxidative stress, and heat shock proteins or chaperones (Aertsen et al., 2004, 2005; Gänzle and Liu, 2015). Remarkably, the protective effect of the LHR on pressure resistance of *E. coli* MG1655 was attributable to the protein homeostasis module in pRF1 (Figures 6, 7). In contrast, while cloning of the full length LHR1 increases heat resistance in *E. coli* more than 1,000,000 fold (Mercer et al., 2015; Boll et al., 2017), cloning of the LHR-encoded heat shock proteins has little effect on heat resistance of *E. coli* (Mercer R. M. et al., 2017; Mercer R. et al., 2017) and increases heat resistance of *Pseudomonas aeruginosa* and *Klebsiella pneumoniae* only 100 fold (Bojer et al., 2010; Lee et al., 2018). The strain dependent and differential effect on pressure and heat resistance provides an excellent tool to probe the relationship between protein aggregation and stress resistance in *E. coli*.

### Effects of LHR on Protein Aggregation *in vivo*

Heat shock proteins are universally present in gram-positive and gram-negative bacteria and prevent heat-induced protein aggregation (Jakob et al., 1993; Lee et al., 1997; Kerner et al., 2005). ClpK_GI_, an ATP-dependent HSP100 family protein, co-operates with DnaK and other heat shock proteins to reverse protein aggregation in *E. coli* in an ATP-dependent manner (Mogk et al., 2003; Zolkiewski, 1999). The sHsp20 from *P. aeruginosa* exhibited *in vitro* chaperone activity (Lee et al., 2015); ClpK_GI_ (ClpG_GI_ in *P. aeruginosa*) is a stand-alone disaggregase with ATPase activity that dissolves heat-induced protein aggregates (Lee et al., 2018). Disaggregase activity of ClpG_GI_ was demonstrated *in vivo* in ClpG_GI_ deficient strains and complements (Lee et al., 2018). ClpG_GI_ complements the DnaK-ClpB mediated disaggregase activity that is encoded in the core genome of *P. aeruginosa* (Lee et al., 2018). We confirmed and expanded these observations by proteomic and microscopic observation of protein aggregates *in vivo* (Stewart et al., 2005; Lindner et al., 2008), by demonstrating activity against pressure-induced protein aggregates, and by relating protein aggregation to the heat and pressure resistance of *E. coli*. Cloning of the three heat shock proteins sHsp20, ClpK_GI_, and sHSP, was sufficient to confer the phenotype related to disaggregation in heat- and pressure treated *E. coli*; however, these three proteins do not confer heat or chlorine resistance in *E. coli* (Mercer R. M. et al., 2017; Mercer R. et al., 2017; Wang et al., 2020). Moreover, expression of sHsp20, ClpK_GI_, and sHSP in *E. coli* LMM1010 reduced protein aggregation but did not increase the pressure resistance of this strain (this study). The function of YdfX1 and YdfX2 remains to be elucidated but likely pertains to envelope stress (Mercer R. et al., 2017). The protease DegP may function to eliminate misfolded or damaged proteins in the periplasm (Baneyx and Mujacic, 2004; Krojer et al., 2008; Gayán et al., 2019). Because protein aggregation differs even between individual cells in clonal populations, protein aggregates were analyzed by fluorescence microscopy (Mortier et al., 2019). Alternative methods for determination of *in vivo* protein aggregation may be useful, however, to further delineate the contribution of chaperons and disaggregases on stress resistance in *E. coli* (Roy et al., 2014).

## Conclusion

Locus of heat resistance positive *E. coli* include pathogenic strains (Mercer et al., 2015; Ma and Chui, 2017) and were identified in chlorine treated wastewater and in food. Control of heat and chlorine resistant *E. coli* in the food and water supply is facilitated by improved understanding of the expression and functions of LHR-encoded proteins. The 11 LHR proteins that are expressed in *E. coli* not only reduce protein aggregation (Lee et al., 2015, 2018; this study) but also relate to envelope stress, oxidative stress, and osmotolerance, and thus provides multi-faceted protection against heat. Functions related to protein aggregation provide cross-protection against hydrostatic pressure (this study); the multiple protective functions encoded by the LHR also confer resistance to other stressors including oxidative stress (Kitagawa et al., 2000; Zhi et al., 2016; Wang et al., 2020).

## Data Availability Statement

The datasets generated for this study can be found in the PRIDE partner repository, PXD011023.

## Author Contributions

HL, JB, and SH conducted the experiments. HL, MG, RM, and RV contributed to the experimental design. HL and MG wrote the manuscript. HL, RM, JB, SH, LM, RV, and MG contributed to the interpretation of experiments and drafting/editing of the manuscript.

## Conflict of Interest

The authors declare that the research was conducted in the absence of any commercial or financial relationships that could be construed as a potential conflict of interest.

## Footnotes

1 http://proteomecentral.proteomexchange.org
