# Supplementary material for: Heat and Pressure Resistance in Escherichia coli Relates to Protein Folding and Aggregation
Source: Front Microbiol. 2020 Feb 4;11:111. doi: 10.3389/fmicb.2020.00111 (PMC7010813; doi:10.3389/fmicb.2020.00111)
Supplement: Supplementary file 1 [file Data_Sheet_1.PDF]

Online supplementary material

Heat and pressure resistance in *Escherichia coli* relates to protein folding and aggregation

Hui Li[ab)], Ryan Mercer[a)], Jürgen Behr[cf)], Stephanie Heinzlmeir[c)], Lynn M. McMullen[a)], Rudi F. Vogel[d)], and Michael G. Gänzle[ae*)]

[a)]University of Alberta, Department of Agricultural, Food and Nutritional Science, Edmonton, Canada

[b)]Institute of Quality Standard & Testing Technology for Agro-Products, Chinese Academy of Agricultural Sciences, Key Laboratory of Agro-food Quality and Safety, Ministry of Agriculture, Beijing, China

[c)]Technical University of Munich, Bavarian Center for Biomolecular Mass Spectrometry, Freising, Germany

[d)]Technical University of Munich, Lehrstuhl für Technische Mikrobiologie, Freising, Germany

[e)]Hubei University of Technology, College of Bioengineering and Food Science, Wuhan, P.R. China.

[f)]Leibniz-Institute for Food Systems Biology at the Technical University of Munich, Freising, Germany

Table S1. Result of enrichment analysis of proteins overrepresented in whole cell extracts and inclusion bodies of untreated E. coli MG1655 ibpA-yfp (pLHR), and of the same strain after treatment with 400 MPa for 3 min.

Table S2. Differential enumeration of cells containing none, one, two, or more than two foci representing protein aggregates in *E. coli* LMM1010 *ibpA-yfp* (pRK767) and *E. coli* LMM1010 *ibpA-yfp* (pLHR).

Table S3. Differential enumeration of cells containing none, or one or more foci representing protein aggregates in *E. coli* LMM1010 *ibpA-yfp* (pRK767) and *E. coli* LMM1010 *ibpA-yfp* (pLHR).

Figure S1. Contribution of the locus of heat resistance (LHR) to pressure resistance of *E. coli* LMM1010 and LMM1010 *ibpA-yfp*. The strains carrying the vector of pRK767 (white, LMM1010; gray, LMM1010 *ibpA-gfp*) or the derivatives of this vector with the full length LHR (dark gray, LMM1010; black, LMM1010 *ibpA-yfp*) were treated at 400 MPa at 20 ^{o}C for 1 or 3 min.

Table S1. Result of enrichment analysis of proteins overrepresented in whole cell extracts and inclusion bodies of untreated *E. coli* MG1655 *ibpA-yfp* (pLHR), and of the same strain after treatment with 400 MPa for 3 min.

Overrepresented in whole cell extracts	Overrepresented in inclusion bodies
**Untreated cells**	
Cytosol	Plasma membrane
Protein synthesis	Integral component of membrane
Protein Metabolism	Integral component of plasma membrane
Signal transduction mechanisms, translation, ribosomal structure and biogenesis	Transport and binding proteins
Translation	Cell outer membrane
Structural constituent of ribosome	Defense mechanisms
Cytosolic large ribosomal subunit	Respiration
rRNA binding	Intracellular
Biosynthesis of cofactors, prosthetic groups, and carriers	Inorganic ion transport and metabolism
Amino acid biosynthesis	General function prediction only replication, recombination and repair
Outer membrane-bounded periplasmic space	Cell cycle control, cell division, chromosome partitioning
Cytoplasm	Cell motility
Cytosolic small ribosomal subunit	Cell wall, membrane, envelope biogenesis
Purines, pyrimidines, nucleosides, and nucleotides	Amino acid transport and metabolism energy production and conversion
Nucleosides and Nucleotides	Inorganic ion transport and metabolism post translational modification, protein turnover, chaperones
Cell motility nucleotide transport and metabolism	Transcription factor activity, sequence-specific DNA binding
Zinc ion binding	Function unknown secondary metabolites biosynthesis, transport and catabolism
Stress Response	Regulatory functions
	Plasma membrane respiratory chain complex I
	Membrane
	NADH dehydrogenase complex
**Pressure treated cells**	
Plasma membrane	RNA metabolism
	Signal transduction mechanisms, translation, ribosomal structure and biogenesis

Table S2. Differential enumeration of cells containing none, one, two, or more than two foci representing protein aggregates in *E. coli* LMM1010 *ibpA-yfp* (pRK767) and *E. coli* LMM1010 *ibpA-yfp* (pLHR). Data are based on triplicate independent experiments with observation of 100 cells per replicate.

Number of protein aggregates (foci) per cell	% of cells of *E. coli* LMM1010 *ibpA-yfp* harbouring a different number of fluorescent foci	
	pRK767	pLHR
0	0.6 ± 0.5	10.9 ± 7.2
1	97.6 ± 1.5	70.1 ± 5.7
2	1.7 ± 1.5	6.3 ± 0.9
≥ 3	0.1 ± 0.2	12.6 ± 4.1

Table S3. Differential enumeration of cells containing none, or one or more foci representing protein aggregates in *E. coli* LMM1010 *ibpA-yfp* (pRK767) and *E. coli* LMM1010 *ibpA-yfp* (pLHR). Shown is the percentage of cells with foci after treatment at 60 °C for 5 min or at 400 MP for 3 min. Data are based on triplicate independent experiments with observation of 100 cells per replicate.

Foci	pRK767	pLHR
% of cells with or without protein aggregates after treatment at 60 °C		
One or more	69.9 ± 11.3	37.4 ± 9.4
None	30.1 ± 11.3	62.6 ± 9.4
% of cells with or without protein aggregates after treatment at 400 MPa		
One or more	83.7 ± 2.2	50.7 ± 2.8
None	16.3 ± 2.2	49.3 ± 2.8

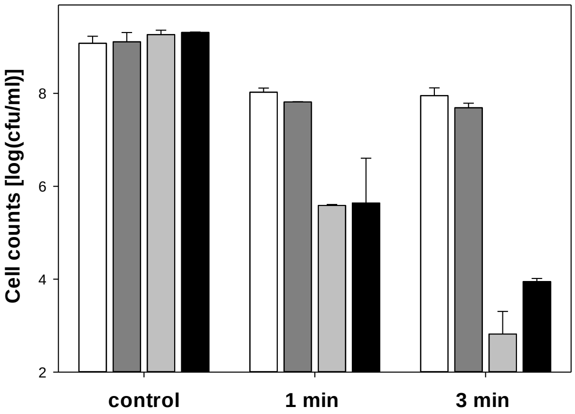

Figure S1. Contribution of the locus of heat resistance (LHR) to pressure resistance of *E. coli* LMM1010 and LMM1010 *ibpA-yfp*. The strains carrying the vector of pRK767 (white, LMM1010; gray, LMM1010 *ibpA-gfp*) or the derivatives of this vector with the full length LHR (dark gray, LMM1010; black, LMM1010 *ibpA-yfp*) were treated at 400 MPa at 20 ^{o}C for 1 or 3 min. Experiments were replicated three times.
